# Sunitinib prevents cachexia and prolongs survival of mice bearing renal cancer by restraining STAT3 and MuRF-1 activation in muscle

**DOI:** 10.18632/oncotarget.2812

**Published:** 2014-11-15

**Authors:** Francesca Pretto, Carmen Ghilardi, Michele Moschetta, Andrea Bassi, Alessandra Rovida, Valentina Scarlato, Laura Talamini, Fabio Fiordaliso, Cinzia Bisighini, Giovanna Damia, Maria Rosa Bani, Rosanna Piccirillo, Raffaella Giavazzi

**Affiliations:** ^1^ Department of Oncology, IRCCS-Istituto di Ricerche Farmacologiche “Mario Negri”, 20156 Milan, Italy; ^2^ Department of Phisics, Politecnico di Milano, 20133 Milan, Italy; ^3^ Department of Cardiovascular Research, IRCCS-Istituto di Ricerche Farmacologiche “Mario Negri”, 20156 Milan, Italy; ^4^ Present address: Philochem AG, 8112 Otelfingen, Switzerland

**Keywords:** renal carcinoma, cancer cachexia, muscle wasting, sunitinib, STAT3, xenograft model

## Abstract

Tyrosine kinase inhibitors, affecting angiogenesis, have shown therapeutic efficacy in renal cell carcinoma (RCC). The increased overall survival is not fully explained by their anti-tumor activity, since these drugs frequently induce disease stabilization rather than regression. RCC patients frequently develop cachectic syndrome. We used the RXF393 human renal carcinoma xenograft that recapitulates the characteristics of the disease, including the growth in the mouse kidney (orthotopic implantation), and the induction of cachexia with subsequent premature death. Sunitinib prevents body weight loss and muscle wasting and significantly improves the survival of RXF393-bearing nude mice. The anti-cachectic effect was not associated to direct anti-tumor activity of the drug. Most relevant is the ability of sunitinib to reverse the cachectic phenotype and rescue the animals from the loss of fat tissue. Body weight loss is prevented also in mice bearing the C26 colon carcinoma, classically reported to induce cachexia in immunocompetent mice. Among the mechanisms, we herein show that sunitinib is able to restrain the overactivation of STAT3 and MuRF-1 pathways, involved in enhanced muscle protein catabolism during cancer cachexia.

We suggest that off-target effects of angiogenesis inhibitors targeting STAT3 are worth considering as a therapeutic option for patients who develop cachexia, independently of their anti-tumor activity.

## INTRODUCTION

Cachexia is a dramatic wasting syndrome associated with several chronic diseases, including cancer, and primarily involves loss of muscle mass [1]. Cancer cachexia affects up to 80% of cancer patients and causes reduced physical function, low tolerance to anti-cancer therapy and shorter survival [1, 2]. Muscle wasting results from excess of protein catabolism over synthesis and during cancer it is triggered by increased levels of proinflammatory cytokines [3, 4]. Given the complexity of this syndrome, progress in the treatment of cancer cachexia has been slow. Cancer cachexia is an important unmet medical need for which multimodal management is normally aimed at the best supportive care [5, 6].

Patients afflicted by renal cell carcinoma (RCC) frequently develop cachexia [7]. This syndrome is believed to be caused by the secretion of cytokines or hormones from the tumor or the immune system [8]. Tumor-specific growth factors, like vascular endothelial growth factor (VEGF) and platelet-derived growth factor (PDGF), are also highly secreted by RCC [8]. In the past five years, the introduction of targeted anti-angiogenic therapy has dramatically enlarged the number of therapeutic options for the treatment of RCC and significantly improved the prospects for patients [9].

Sunitinib is a multi-targeted receptor tyrosine kinase inhibitor (TKI) that mainly targets VEGF and PDGF receptors and it was the first oral TKI to gain regulatory approval in this setting [10]. Sunitinib is the reference standard of care, recommended in international guidelines for the first-line treatment of favorable- or intermediate-risk RCC [11]. The range of agents available for the treatment of RCC has expanded substantially, including sorafenib, temsirolimus, everolimus, bevacizumab in combination with interferon-α and, more recently, pazopanib and axitinib [12]. However, while increasing overall survival, these targeted drugs frequently induce only disease stabilization. Since survival benefits in RCC patients do not always correlate with tumor response, other unknown mechanisms may account for the clinical benefits.

In the present study, we show that mice bearing RCC-derived RXF393 develop cachexia (i.e. body weight loss), which is prevented and/or reversed upon treatment with sunitinib, resulting in increased survival. Both muscle and fat tissues undergo wasting in RXF393-bearing mice and sunitinib is able to prevent the loss of both. This unprecedented therapeutic effect is not associated with its anti-tumor activity and involves prevention of STAT3 activation and MuRF-1 over-expression in muscles. As a consequence, STAT3 inhibitors should be worth evaluating as a therapeutic option for cancer patients who develop cachexia.

## RESULTS

### Sunitinib prevents RXF393-induced cachexia

**Figure 1:**
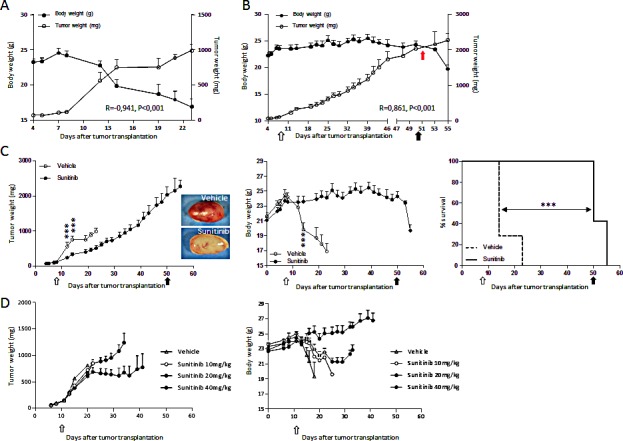
RXF393-induced cachexia is prevented by sunitinib treatment independently from tumor inhibition A, RXF393 cells were injected subcutaneously into the flank of nude mice: tumor and body weight are plotted over time and their correlation is shown. B, RXF393-bearing mice were randomized (TW 120 mg, n=7/group) to receive sunitinib (40 mg/kg, p.o.) daily for the indicated time (arrows): tumor and body weights are reported and their correlation is shown. C, RXF393-bearing mice were randomized (TW 120 mg, n=7/group) to receive sunitinib or vehicle daily for the indicated time (arrows). Tumor weights (left panel) and representative images (inserts) of the gross appearance of treated and untreated tumors, excised when mice were euthanized, are shown. Body weights are plotted over time (middle panel). Mice treated with sunitinib or not were sacrificed when they lost 20% of body weight and/or showed signs of distress (loss of mobility, kyphosis, tremor). Survival curves are depicted (right panel). D, RXF393-bearing mice were randomized (TW 150 mg, n=9/group) to receive vehicle or sunitinib at the doses of 10, 20 or 40mg/kg daily p.o. The effects of sunitinib on tumor growth (left panel) and BW (right panel) are reported. Open arrow=treatment starts, full arrow=treatment ends, red arrow=day of inversion.

The human renal carcinoma RXF393 transplanted in nude mice forms a rapidly growing tumor causing progressive body weight loss (BWL), typical of the cachectic syndrome. As shown in Figure 1A, all RXF393-bearing mice rapidly lost weight (a hallmark of cachexia) around 15 days after subcutaneous tumor transplantation, when tumor weight (TW) was about 700 mg, and died shortly after. A significant inverse correlation was observed between tumor and body weight (R=-0.941, P<0.001). When tumor-bearing mice were treated with sunitinib, the inverse correlation between tumor and body weight was lost, but it was rapidly restored when treatment was interrupted (Fig.1B). Sunitinib treatment (started at about 100 mg of TW) had a moderate, but significant anti-tumor effect (T/C 30%) (Fig.1C, left). All the RXF393-bearing mice treated with vehicle developed cachexia (BWL > 20%) and ought to be killed within 23 days after tumor transplantation (Fig.1C, middle), whereas the overall survival (time of sacrifice) of sunitinib-treated mice was significantly higher (MST 51 days; ILS 250%) (Fig.1C, right) and, surprisingly, the tumor reached the maximal ethically accepted weight (< 2000mg) without causing cachexia. At autopsy, subcutaneous tumors harvested from sunitinib-treated mice showed a pale phenotype, compared to the highly vascularized appearance of the vehicle-treated tumors, consistent with the anti-vascular activity of sunitinib (Fig.1C, inserts). Immunohistological analysis confirmed the lower vessel density in sunitinib-treated tumors (data not shown).

To exclude that the anti-cachectic effects of sunitinib depends on its effect on tumor growth, we compared the therapeutic active dose of sunitinib (40mg/kg) to sub-optimal doses (20 and 10mg/kg) in RXF393-bearing mice. As shown in Fig.1D, left, 40mg/kg inhibited tumor growth, while doses of 20mg/kg or 10mg/kg did not exert anti-tumor effects. However, a dose-response effect on BWL was observed: 10mg/kg delayed BWL by four days and 20mg/kg was able to preserve body mass (Fig.1D, right). These results suggest that the effect on cachexia by sunitinib involves mechanisms aside from inhibition of angiogenesis and tumor growth.

**Figure 2:**
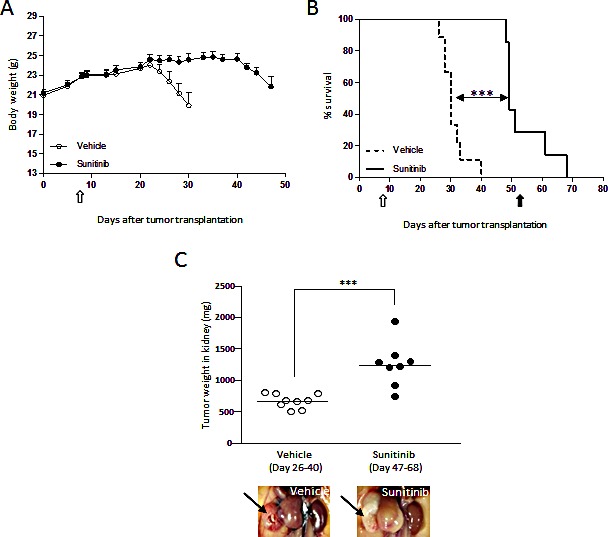
Cachexia is prevented by sunitinib treatment in the RXF393 orthotopic model RXF393 cells were injected orthotopically into the right kidney of nude mice and growing tumors were treated with sunitinib (40 mg/kg, p.o.) or vehicle daily for the indicated time (n=9/group). A, Body weights are plotted over time. B, Mice treated with sunitinib or not were sacrificed when they lost 20% of body weight and/or showed signs of distress (loss of mobility, kyphosis, tremor) and survival curves are shown. C, Tumor weights were determined at sacrifice and median is reported. Range day of sacrifice is reported in brackets. Representative images of the gross appearance of treated and untreated tumors growing in the kidney are shown in the inserts. Open arrow=treatment starts, full arrow=treatment ends.

To confirm the anti-cachectic effects of sunitinib in a more relevant setting, RXF393 was transplanted orthotopically in the kidney of nude mice (Fig.2). Tumor-bearing mice started losing body weight in three weeks (Fig.2A), and were to be sacrificed (MST 30 days, Fig.2B) when their tumorgrafts reached about 600 mg (Fig.2C), because of clear signs of discomfort (i.e. kyphosis, immobility, tremor; 20% of BWL). In contrast, sunitinib-treated mice lived significantly longer (MST 51 days; ILS 70%, Fig.2B) and no sign of BWL was observed when mice were sacrificed with a tumor mass of approx. >1g, (Fig.2A and C). At autopsy, tumors growing in the kidney of sunitinib-treated mice had a pale phenotype, consistent with the drug's activity (Fig.2C).

### Sunitinib prevents RXF393-induced muscle wasting

One of the key features of cancer-induced wasting syndrome is the loss of skeletal muscle mass [13]. To test whether cachexia was accompanied by skeletal muscle atrophy, and whether sunitinib prevented this, mice bearing subcutaneous RXF393 were treated with sunitinib or vehicle and muscle wasting was analyzed at autopsy. To exclude differences due to the tumor burden, RXF393-bearing mice from vehicle- and sunitinib-treated groups were sacrificed when tumor reached comparable sizes.

**Figure 3:**
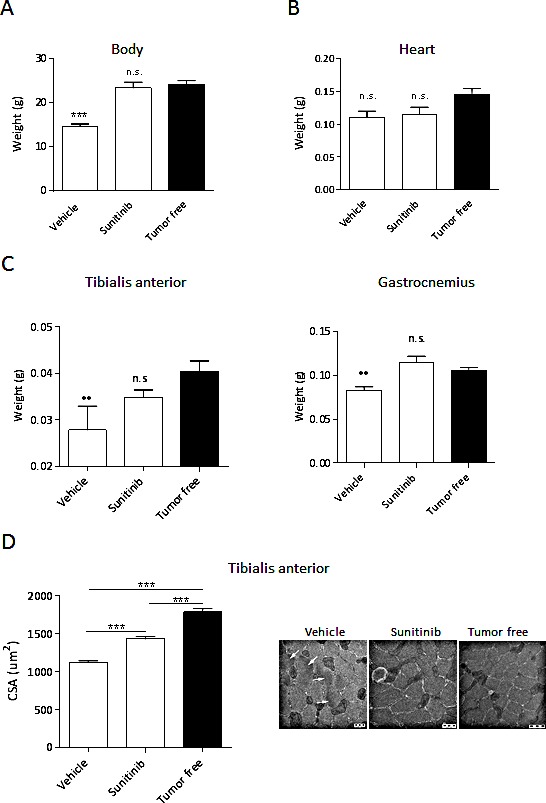
Sunitinib prevents RXF393-induced skeletal muscle wasting RXF393-bearing mice were randomized (TW 120 mg, n=7/group) to receive sunitinib (40 mg/kg p.o.) or vehicle daily. Animals were sacrificed at tumor sizes of about 400mg, and bodies (A), hearts (B), TA and gastrocnemius muscles (C) were weighed. D, The effects of treatment on the TA muscles were also determined by quantitation of the CSA. Representative ultrastructural images by electron microscopy, showing the morphology of the TA in the various conditions are provided. Results are plotted as mean ±SD. n.s.=not significant.

Comparable tumor weights (about 400mg) were associated to significant BWL in vehicle-treated mice, whereas the body weight of sunitinib-treated ones were undistinguishable from age- and sex-matched healthy mice (tumor-free) (Fig.3A). After death, heart, tibialis anterior (TA) and gastrocnemius muscles were collected and weighed. In RXF393-bearing mice, no loss of heart weight was observed both in vehicle- and sunitinib-treated mice (Fig.3B) compared to healthy mice, while the weight of TA and gastrocnemius muscles from vehicle-treated ones decreased by about 25% (Fig.3C). Strikingly, the mean weight of both muscles from sunitinib-treated mice did not significantly differ from that of healthy mice, indicating that sunitinib completely protects from RXF393-induced muscle wasting (Fig.3C). Moreover, the cross-sectional area (CSA) of the TA myofibers showed a significant reduction between vehicle-treated and healthy animals (1122 μm^2^ and 1789 μm^2^, respectively), which was partially but significantly restored upon sunitinib treatment (1432 μm^2^) (Fig.3D, left). Accordingly, electron microscopy ultra-structural analysis of the TA muscles of healthy mice showed the expected cross-sectional organization of thin and thick filaments in myofibrils, surrounded by mitochondria and cisternae of the sarcoplasmic reticulum. Conversely, cachectic muscles from tumor-bearing mice showed derangement and degeneration of both types of filaments, while mitochondria and sarcoplasmic reticulum retained their normal morphology. Interestingly, treatment of cachectic mice with sunitinib reversed the abnormal morphology of TA, restoring the normal architecture of sarcomeres (Fig.3D, right).

### Sunitinib prevents C26-induced cachexia

The beneficial effects of sunitinib were further confirmed in another experimental model fully characterized for cancer cachexia and previously used to study anti-cachexia interventions: the murine C26 colon carcinoma [14].

**Figure 4:**
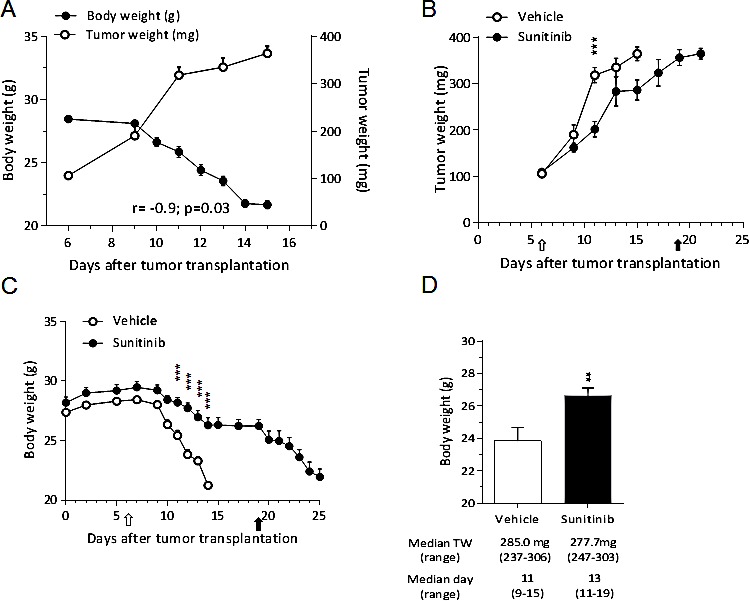
Sunitinib prevents C26-induced cachexia A, C26 cells (1×10^6^) were inoculated subcutaneously in the upper right flank of BALB/c mice. Tumor and body weight are plotted over time and their correlation is shown. B-C, C26-bearing mice were randomized (TW 100 mg) to receive sunitinib (40 mg/kg, p.o.) or vehicle (n=12 /group) daily for the indicated time; tumor growth (B) and body weight (C) are reported. Open arrow = treatment starts, full arrow = treatment ends. D, Body weight of animals with tumors at the same size (approx. 280 mg, irrespectively of the time after tumor transplantation) (n=7/group) is reported. Median tumor weight and day with range are reported.

As expected, the C26 tumor induced about 5% BWL already 11 days after transplantation and, when BWL reached 20%, it led to death 90% of the vehicle-treated mice within 15 days (Fig.4A). Likewise RXF393 model, there was a significant inverse correlation between tumor and body weight (R=-0.9, P<0.05) (Fig.4A).

Sunitinib only minimally inhibited the tumor growth (T/C 69%) (Fig.4B), but it significantly delayed the BWL compared to vehicle-treated mice (Fig.4C, left). However, when the treatment was interrupted at day 20, all the mice previously treated with sunitinib lost weight and were terminally euthanized within one week (Fig.4C).

To exclude that the lower BWL in sunitinib-treated mice was caused by smaller tumors due to treatment, we compared mice bearing tumors of similar size (about 280 mg), irrespectively of the time after transplantation and found that the body weight of sunitinib-treated was significantly higher than that of vehicle-treated mice (Fig.4D).

### Sunitinib reverses RXF393-induced cachexia

**Figure 5:**
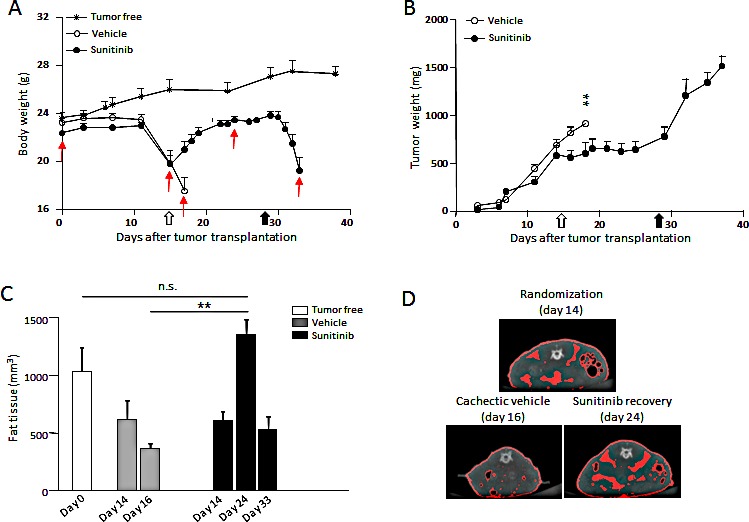
Sunitinib reverses RXF393-induced cachexia Mice bearing subcutaneously RXF393 were randomized with a cachectic phenotype, (10% BWL and initial signs of discomfort) and treated with sunitinib (40mg/kg p.o.) or vehicle daily for two weeks (n=7-9/group). A-B, The effects of sunitinib on body and tumor weights are shown in A and B, respectively. C-D, The effect of sunitinib on fat content was determined by micro-CT scan. A quantitation of abdominal fat at the indicated times is reported (C). Data are plotted as mean±SD (n=5). Representative images from mice at randomization or from vehicle- or sunitinib-treated mice are shown and the fat tissue is highlighted in red (D). Open arrow=treatment starts, full arrow=treatment ends, red arrows=day of CT analysis.

To test the ability of sunitinib to reverse the wasting syndrome once established, mice bearing RXF393 and developing cachexia were randomized to receive sunitinib or vehicle, when BWL was around 10% (14 days after tumor implantation). As shown in Fig.5A, sunitinib completely and rapidly reversed BWL even when treatment started with larger tumors (about 600-700 mg, Fig.5B). The recovery in body weight was accompanied by a gain in fat tissue, as shown by micro-CT (computed tomography) scans of the entire abdominal region of tumor-bearing mice treated with sunitinib (Fig.5C and D, day 24). Vehicle-treated mice lost fat tissue concomitantly to BWL (day 16). As expected, soon after suspension of sunitinib treatment, mice started to lose weight (Fig.5A) and fat tissue (Fig.5C, day 33) at a level comparable to untreated mice. Of note, body mass was not significantly affected in tumor-free mice treated or not with sunitinib (data not shown). Altogether, these data show that sunitinib treatment exerts its effects, such as preservation of body mass and fat tissue, as long as its administration is maintained, and even if cachexia has already advanced.

### Sunitinib prevents MuRF-1 and STAT3 activation in muscle of RXF393-bearing mice

**Figure 6:**
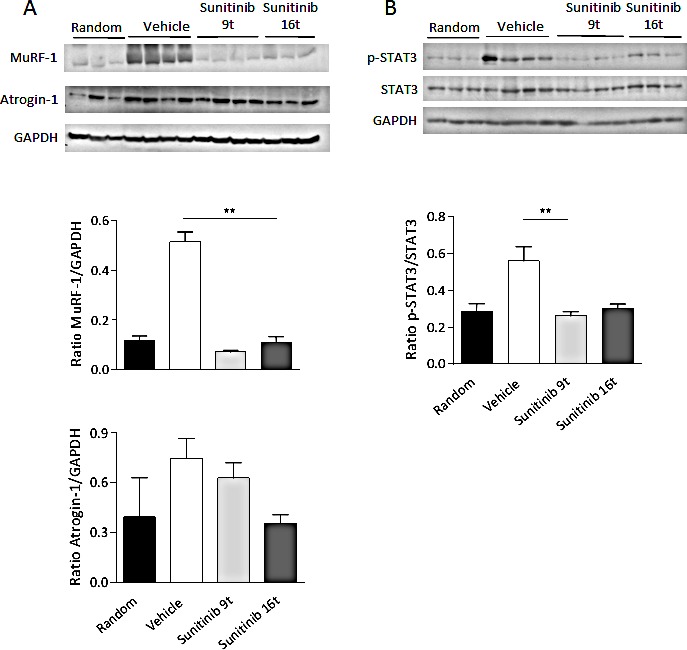
Skeletal muscle wasting is prevented by sunitinib through inhibition of MuRF-1 and p-STAT3 A-B, Tibialis anterior muscles of untreated or sunitinib-treated mice (after 9 (9t) or 16 (16t) days of treatments) were analyzed by Western Blot for MuRF-1, atrogin-1 (A), p-STAT3 and STAT3 (B) protein levels. Tibialis anterior of non-cachectic mice, sacrificed at randomization, were chosen as reference (indicated as Random). GAPDH was used as internal loading control. Densitometric analysis was performed and quantitation of protein levels is provided. Results are plotted as mean ±SD.

Loss of skeletal muscle mass is generally due to reduced protein synthesis, increased degradation or a relative imbalance of the two [3, 4]. The molecular pathways implicated in these mechanisms were investigated in TA muscles of vehicle- and sunitinib-treated mice. The Muscle RING Finger 1 protein (MuRF-1), a muscle-restricted ubiquitin ligase involved in the accelerated protein degradation during various kinds of muscle atrophy [15], was found highly up-regulated in muscles from cachectic RXF393-bearing mice, while sunitinib treatment was able to prevent MuRF-1 up-regulation (Fig.6A). More prolonged sunitinib treatment led to down-regulation of atrogin-1, another muscle-specific ubiquitin ligase triggering muscle atrophy [15, 16], although the effect was less evident. Levels of p-AKT over total AKT, usually considered as index of protein synthesis, were also evaluated, but not significant difference was detected (data not shown).

An important mediator implicated in the development of cancer cachexia is the transcription factor STAT3 that previous work has shown activated by circulating pro-inflammatory cytokines (i.e. IL-6) [17]. Muscles from cachectic RXF393-bearing mice exhibited over-activation of STAT3, as shown by higher levels of phosphorylated STAT3 (p-STAT3) compared to non-cachectic ones (analyzed at randomization) (Fig.6B). Sunitinib inhibited p-STAT3 of muscles from RXF393-bearing mice at levels comparable to non-cachectic mice.

Similar results were obtained with sorafenib, another multitargeted TKI, also used for the treatment of renal cancer [18]. Administration of sorafenib prevented the body and muscle weight loss in RXF393-bearing mice and this was associated with the inhibition of STAT3 and MuRF-1 pathways in TA muscles (Supplementary Fig. 1).

IL-6 was elevated in plasma of mice bearing RXF393 or C26, but sunitinib did not change its levels in neither model (Supplementary Fig.2), suggesting that the effect of sunitinib is not mediated by the direct attenuation of this circulating cytokine.

Overall, these data indicate that RXF393-bearing mice offer a unique model to test drugs against cancer cachexia and that sunitinib, as well as sorafenib, can prevent cachexia *in vivo* by lowering protein catabolism through inhibition of STAT3/MuRF-1 activation at least in muscles.

## DISCUSSION

Renal cell carcinoma is one of the malignancies that mostly causes a cancer-associated systemic syndrome (i.e. cachexia), mainly consisting in progressive loss of the body energy stores and likely reflecting the high production of cytokines and growth factors [7, 8]. Herein, we report that treatment with sunitinib (and sorafenib, Supplementary data) prolonged the survival of mice bearing the human kidney carcinoma RXF393, transplanted either ectopically in the subcutis or orthotopically in the kidney (i.e. mimicking the site of tumor origin), by blocking the BWL (i.e. muscle and fat wasting) caused by cancer growth.

Unexpected was the ability of sunitinib to reverse the cachectic phenotype once established. In fact, sunitinib reversed BWL and rescued the animal from the loss of abdominal fat tissue. The anti-cachectic effects of sunitinib are not associated to reduced tumor growth, as revealed by the prevention of cachexia also at sub-optimal doses of sunitinib that did not inhibit tumor growth. Importantly, BWL was prevented also in a syngeneic tumor model, the C26 colon cancer, classically reported to induce cachexia in immunocompetent mice [19]. This shows that such anti-cachectic effect also occurs with an intact immune system and, furthermore, implies that it is not restricted to a single tumor model. These results might explain the clinical evidence that often targeted drugs, including sunitinib, increase overall survival of RCC patients, without inducing tumor shrinkage, but rather causing disease stabilization [18, 20].

Of note, during human RCC both cancer and subsequent kidney dysfunction may account for the cachectic appearance, further accelerating muscle loss [21]. Our data, showing similar rates of BWL in mice bearing a tumor subcutaneously or orthotopically in the kidney, possibly exclude that kidney failure may worsen the RXF393-related cachexia.

We have investigated the anti-cachectic effect exerted by sunitinib not only at the macroscopic level of multiple tissues (fat, heart and skeletal muscles) but also at the ultrastructural level in skeletal muscle (i.e. the most affected tissue in cancer cachexia). Surprisingly, we observed that sunitinib not only was able to spare the muscle mass but even the misalignment of myofibers induced by cancer progression. As reported by Aulino and coworkers [19] in C26-bearing mice, we report that cachectic muscles from RXF393 mice display an aberrant distribution of thin and thick filaments that may be due to selective degradation of muscle proteins [22]; these aberrations can be prevented by sunitinib, allowing preservation of muscle mass and probably of its function.

Decreased protein synthesis and increased proteolysis are among the mechanisms leading to muscle loss [3]. During atrophy, MuRF-1 and atrogin-1 are the crucial muscle-specific ubiquitin ligases that direct the polyubiquitination of proteins to target them for proteolysis by the 26S proteasome, mediating sarcomeric breakdown (MuRF-1, which degrades myosins) [23] or shifting gene expression towards a less myogenic phenotype (atrogin-1, which degrades MyoD) [24]. Our results indicate that cachexia prevention by sunitinib is mainly determined by the reduction of proteolysis, rather than increased synthesis, as shown by suppression of MuRF-1 and, though less evident, atrogin-1 induction and unchanged levels of p-AKT/AKT ratio. Since FoxO3 is a master transcription factor driving muscle wasting by up-regulating both MuRF-1 and atrogin-1 [25, 26], we have also measured the levels of p-FoxO3 over total FoxO3 in the vehicle and sunitinib-treated muscles, but no difference was detected. Instead, our data suggest that improvement of cachexia is associated to STAT3 inhibition in muscles by sunitinib.

Cancer cachexia is triggered by increased systemic inflammation, where pro-inflammatory cytokines (e.g. IL-6) play a major role [27]. Previous reports demonstrated that STAT3 activation induced by IL-6 is per se sufficient to induce muscle fiber wasting *in vitro* as well as *in vivo* and that STAT3 inhibition would abolish skeletal muscle wasting downstream of IL-6 in cancer cachexia models [17, 28]. Interestingly, STAT3 has been found activated also in wasting muscles from patients suffering from chronic kidney disease [29] and very recently involved in the control of satellite cell expansion and muscle repair [30]. Moreover, the IL-6/STAT3 pathway has been also implied in increased lipolysis of C26-bearing mice [31], thus suggesting that in our experimental models sunitinib may spare fat and muscle tissues through similar targets. We found that circulating IL-6 was elevated in both nude mice bearing RXF393 and in immunocompetent mice bearing C26, (as described by others [32, 33]), but was not reduced by sunitinib (Supplementary Fig.2). This indicates that the anti-cachectic effect of sunitinib is not due to reduced circulating IL-6 or that the inhibitory target of sunitinib is downstream of the IL-6 cascade (i.e. JAK) [17]. Similar results were found upon treatment of cachectic rodents with anti-myostatin inhibitors, where signs of cachexia were reversed without affecting IL-6 or TNF-α plasma levels [32].

The RXF393 xenograft model secretes high levels of VEGF, likely enough to cause a cachectic syndrome [34, 35]. Indeed, Cao et coworkers have shown that systemic treatment with anti-VEGF agents reversed VEGF-induced cancer associated systemic syndrome and prolonged mouse survival [34]. Surprisingly, antibodies anti-VEGF (bevacizumab) or anti-VEGF receptor 2 (DC101) did not protect RXF393-carrying mice from cachexia to the same extent as sunitinib (data not shown). Presumably, other cytokines or growth factors are responsible for cachexia in our tumor model.

Although our understanding of cancer cachexia has improved dramatically in the past few years, guidelines for the prevention and treatment of cancer-related cachexia are lacking. New treatments with myostatin inhibitors, thalidomide, selective COX-2 inhibitors, ghrelin mimetics and selective androgen receptor modulators have shown promising results, but their efficacy need to be confirmed in clinical trials that are at present testing multimodal interventions against cancer cachexia [5, 6].

Overall, we show that TKIs, such as sunitinib, prevent BWL and preserve muscle and fat tissue, by inhibiting STAT3 phosphorylation. STAT3 has been identified as a novel tumor target and several inhibitors are under development [36]. Only very recently, its pharmacological inhibition has been shown to be beneficial against cancer cachexia in experimental models [37]. Our results further encourage to regard STAT3 inhibitors as therapeutic option for cancer patients who develop cachexia, independently of their direct anti-tumor activity.

## MATERIAL AND METHODS

### Cell lines

RXF393 (VHL-WT) kidney carcinoma cell line [33], was obtained from the NCI Tumor Repository and cultured in RPMI1640 (Gibco) with 10% FBS (Sigma Aldrich). Authentication of the cell line was done using the AmpFlSTR® Identifiler® PCR Amplification Kit (Applied Biosystems). Murine C26 colon cancer cells [19] were a kind gift of Mario Paolo Colombo (IRCCS-Istituto Nazionale dei Tumori, Milan) and cultured in DMEM high glucose (Gibco) with 10% FBS and 1mM L-glutamine (Gibco). Stocks of the cell lines were stored frozen in liquid nitrogen, and kept in culture for no more than six passages before injection in mice.

### Mice and tumor models

RXF393 cells were injected subcutaneously (s.c., 1.5×10^6^ cells) into the flank or orthotopically (1×10^5^ cells) into the right kidney of six- to eight-week-old female NCr-nu/nu mice (Harlan), as previously described [38]. Nude mice were maintained under specific pathogen-free conditions and handled using aseptic procedures. C26 cells (1×10^6^) were inoculated s.c. in the upper right flank of male BALB/c mice (Harlan).

Tumor growth s.c. was measured twice a week with Vernier Caliper and plotted as mean tumor weight (±SE) against days after tumor transplantation. The efficacy of treatment was expressed as best tumor growth inhibition [%T/C = (median weight of treated tumors/median weight of control tumors) × 100]. In the orthotopic model, tumor appearance was checked twice a week by palpation. Cachexia was followed by recording body weight that was plotted as the mean (±SE) against days after tumor transplantation. Mice were sacrificed when BWL reached > 20% and/or the animals showed clear signs of distress (ruffled fur for BALB/c mice, tremor, loss of mobility, kyphosis), however with a tumor burden < 2 g. Survival day was the time at which mice were euthanized. The increment of life span (ILS) was calculated as 100x[(median survival day of treated mice-median survival day of vehicle treated mice)/ median survival day of vehicle treated]. Animal study management and data collection were done with the Study Director 1.8 software (Studylog System, Inc., San Francisco, USA) connected to a digital caliper and an electronic balance.

Procedures involving animals and their care were conducted in conformity with institutional guidelines in compliance with national (Legislative Decree n. 26, March 4, 2014; Authorization n.19/2008-A issued March 6, 2008, by the Italian Ministry of Health) and international laws and policies (EEC Council Directive 2010/63, August 6, 2013; Standards for the Care and Use of Laboratory Animals, U.S. National Research Council, Statement of Compliance A5023-01, October 28, 2008), and in line with Guidelines for the welfare and use of animals in cancer research [39].

### Drugs and reagents

Sunitinib (Chemietek) was dissolved in methocel 0.5% and further diluted in saline solution immediately before use. It was administered daily orally by gavage at the dose of 40mg/Kg, unless otherwise indicated.

### Micro-CT analysis

Mice were anesthetized with a continuous flow of 3% isoflurane/oxygen mixture and positioned prone with both legs at right angles. The region spanning the entire torso to the distal tibia of each mouse was scanned with Explore Locus micro-CT scanner (GE Healthcare) without contrast agents. Micro-CT projections of the animals were acquired using 80 kV, 450 μA current, with 100 ms acquisition time. Four hundred projections were acquired over 360°. The resolution of the acquired images was 93 μm. The reconstructed 3D images were visualized and analyzed using MicroView analysis software (GE Healthcare).

The amount of adipose tissue was determined as described by Luu et al.[40]. Briefly, the gray-scale histogram of the reconstructed images presents a peak that indicates the presence of fat. A low and a high gray-scale threshold corresponding to that peak were chosen. The fat was quantified as the sum of the volumes of all the voxels with a gray-scale value ranging between the low and the high thresholds. The analysis was done on the abdominal region (between the proximal end of L1 and the distal end of S1).

### Electron microscopy

Tibialis Anterior muscles from control, untreated and sunitinib-treated mice were excised at sacrifice, cut in the sagittal plane with a razor blade and fixed with 4% paraformaldehyde (PFA) and 2% glutaraldehyde in phosphate buffer 0.12 M, pH 7.4 for 2 hours at 4°C, followed by incubation at room temperature for 2h in OsO4. After dehydration, tissue samples were cleared in propylene oxide, embedded in epoxy medium (Epon 812 Fluka) and polymerized at 60°C for 72 h. From each sample, ultrathin (60 nm thick) sections of areas of interest were obtained, counterstained with uranyl acetate and lead citrate, and examined with an Energy Filter Transmission Electron Microscope (EFTEM, ZEISS LIBRA® 120) equipped with a YAG scintillator slow-scan CCD camera.

### Muscle fiber cross-sectional area measurements

Eight-μm depth cross-sectional tissue sections from the TA muscle were fixed in cold acetone, stained with hematoxylin and eosin solutions (Fluka) and digitally imaged with a CKX41 microscope (Olympus). For each muscle, at least 8 randomly selected 20X magnification images were quantified with Cell^F^ software (Olympus).

### Western Blot analysis

Proteins from TA muscles were extracted with a lysis buffer supplemented with proteases inhibitor cocktail (Roche), quantitated by BCA Protein or Coomassie Plus Assay Kit (Pierce), separated by SDS-PAGE and transferred to a PVDF membrane (Millipore). Membranes were probed with mouse anti-MuRF-1 (1:30), rabbit anti-atrogin-1 (1:2000, a kind gift of Dr. S. Lecker from Beth Israel Deaconess Medical Center in Boston, USA), rabbit anti-phospho STAT3 (Tyr705, 1:2000, Cell Signaling), mouse anti-STAT3 (1:1000, Cell Signaling) and mouse anti-GAPDH (1:40000, Sigma Aldrich) antibodies. The signal was detected with CDP-Star® Substrate (Life Technologies). The MuRF-1 antibody was raised against rat MuRF-1, as previously described [15].

### Statistical analysis

Statistical analyses were done using Prism Software (GraphPad Prism 5.01). Differences in survival were analyzed by the log-rank test. Correlation was assessed by a standard Pearson correlation, after the symmetry of the distribution was assured with Skewness and Kurtosis. Differences among groups of non-time-related measurements were assessed by one-way ANOVA followed by the Bonferroni post-test.

In the figures, significance is indicated as follows: * P<0.05, ** P<0.01, *** P<0.001.

## SUPPLEMENTARY RESULTS FIGURES



## Abbreviations

RCC: renal cell carcinoma
TKI: tyrosine kinase inhibitor
MuRF-1: Muscle RING Finger-1 protein
BW: body weight
BWL: body weight loss
TW: tumor weight
MST: median survival time
ILS: increment of life span
TA: tibialis anterior
CSA: cross-sectional area
p-STAT3: phosphorylated STAT3
IL-6: interleukin-6
VEGF: vascular endothelial growth factor
TNFα: tumor necrosis factor α.
