# Correction: Sunitinib prevents cachexia and prolongs survival of mice bearing renal cancer by restraining STAT3 and MuRF-1 activation in muscle

**DOI:** 10.18632/oncotarget.9971

**Published:** 2016-06-20

**Authors:** Francesca Pretto, Carmen Ghilardi, Michele Moschetta, Andrea Bassi, Alessandra Rovida, Valentina Scarlato, Laura Talamini, Fabio Fiordaliso, Cinzia Bisighini, Giovanna Damia, Maria Rosa Bani, Rosanna Piccirillo, Raffaella Giavazzi

Present: Due to an error made during the manuscript revision process, a notice designating Drs. Rosanna Piccirillo and Raffaella Giavazzi as joint last authors has been omitted.

Corrected: Correct author list is provided below. Authors sincerely apologize for this oversight.

Original article: Oncotarget. 2015; 6(5): 3043-54. doi: 10.18632/oncotarget.2812.

Francesca Pretto^1,4,*^, Carmen Ghilardi^1,*^, Michele Moschetta^1^, Andrea Bassi^2^, Alessandra Rovida^1^, Valentina Scarlato^1^, Laura Talamini^1^, Fabio Fiordaliso^3^, Cinzia Bisighini^3^, Giovanna Damia^1^, Maria Rosa Bani^1^, Rosanna Piccirillo^1,**^ and Raffaella Giavazzi^1,**^

^1^ Department of Oncology, IRCCS-Istituto di Ricerche Farmacologiche “Mario Negri”, 20156 Milan, Italy

^2^ Department of Phisics, Politecnico di Milano, 20133 Milan, Italy

^3^ Department of Cardiovascular Research, IRCCS-Istituto di Ricerche Farmacologiche “Mario Negri”, 20156 Milan, Italy

^4^ Present address: Philochem AG, 8112 Otelfingen, Switzerland

^*^ These authors have contributed equally to this work

^**^ Joint last authors

