# Supplementary material for: Sunitinib prevents cachexia and prolongs survival of mice bearing renal cancer by restraining STAT3 and MuRF-1 activation in muscle
Source: Oncotarget. 2014 Nov 15;6(5):3043–54. doi: 10.18632/oncotarget.2812 (PMC4413636; doi:10.18632/oncotarget.2812)
Supplement: Supplementary file 1 [file oncotarget-06-3043-s001.pdf]

# Sunitinib prevents cachexia and prolongs survival of mice bearing renal cancer by restraining STAT3 and MuRF-1 activation in muscle

## Supplementary Results

### Sorafenib prevents RXF393-induced cachexia and inhibits STAT3 and MuRF-1 activation

Similarly to sunitinib, sorafenib -another receptor TKI used for the treatment of RCC- prevented BWL, increasing the overall survival of RXF393-bearing mice (Supplementary Fig. 1A). To exclude differences due to the tumor burden, RXF393-bearing mice from vehicle and sorafenib or sunitinib-treated groups were killed when their tumors reached approximately 400 mg. Sorafenib was able to prevent body and TA weight loss to the same extent as sunitinib, while no effect on heart weight was reported with any of the treatments (Supplementary Fig. 1B). Consistently, complete inhibition of MuRF-1 induction and STAT3 activation (represented by p-STAT3 levels) was observed in TA muscles of sorafenib-treated mice (Supplementary Fig. 1C).

### Sunitinib does not alter circulating levels of IL-6 and VEGF

Interleukin-6 and VEGF were measured in plasma samples from RXF393-bearing mice, treated or not with sunitinib. We show that RXF393 xenografts released human IL-6 and VEGF in plasma. The levels were not significantly altered after sunitinib treatment (Supplementary Fig. 2A). Similarly, as previously reported, high levels of murine IL-6 were measured in plasma from mice with C26-tumor, but the levels were not significantly diminished by sunitinib (Supplementary Fig. 2B).

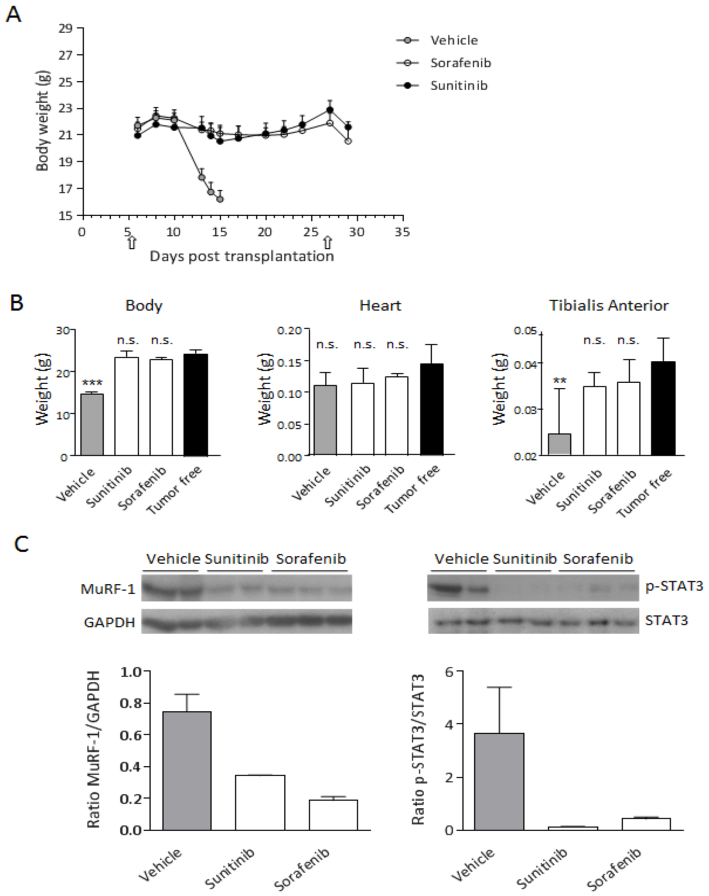


**Supplementary Figure 1: RXF393-induced cachexia is prevented by sorafenib.** RXF393 tumor cells were injected subcutaneously in nude mice and, when tumors reached the mean of 120 mg, mice were randomized to receive daily oral doses (30mg/kg) of sorafenib (Chemietek, dissolved in cremophorEL: ethanol, ratio 1:1 and diluted in saline) or sunitinib (40mg/kg, p.o., reference drug).

**A,** Body weight of vehicle, sorafenib- and sunitinib-treated mice are plotted over time (n=8/group).

**B,** Mice were killed when their tumor weights reached approximately 400mg, and body, heart and TA muscle weight were recorded. Results are plotted as mean ±SD. (n= 4/group). n.s=not significant

**C,** TA muscles from vehicle, sorafenib or sunitinib-treated mice were analysed by Western Blot for MuRF-1 and p-STAT3 protein levels. GAPDH was used as internal reference. Densitometric analysis was performed and quantitation of protein levels is provided. Results are plotted as mean ±SD.

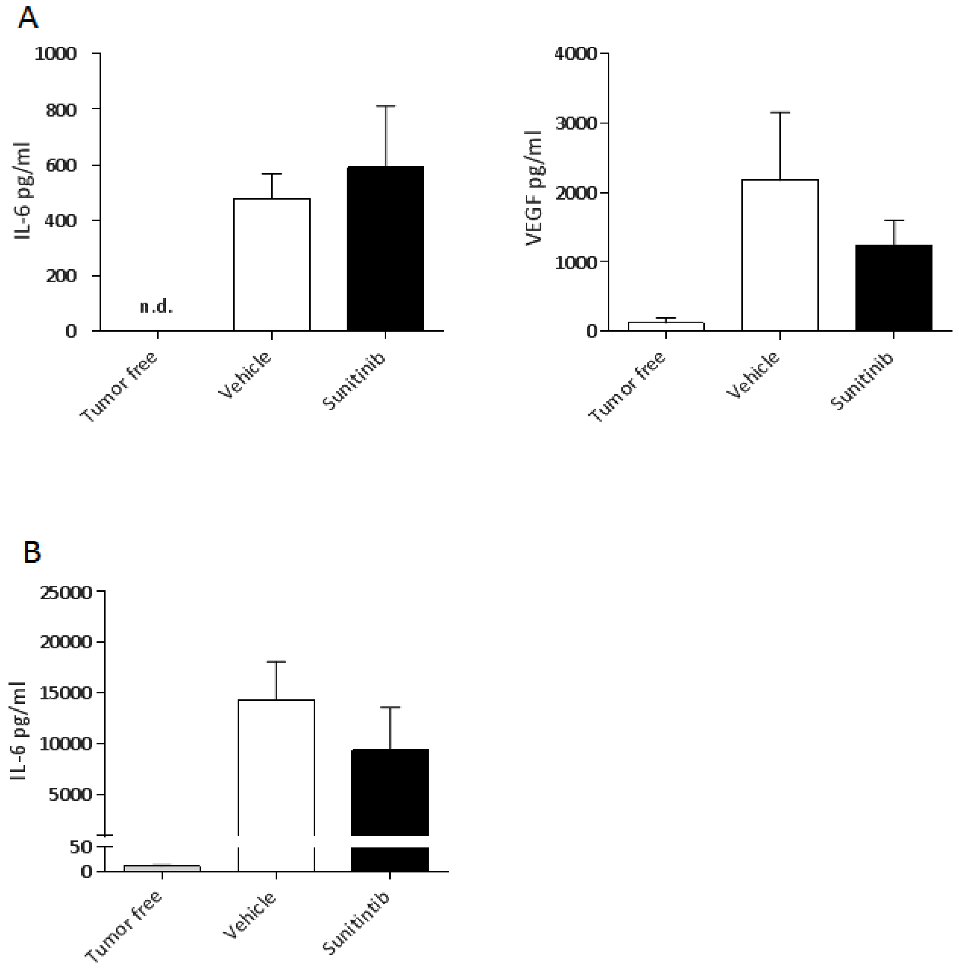


**Supplementary Figure 2: IL-6 and VEGF levels are not altered by sunitinib treatment**

**A**, Human IL-6 and VEGF protein levels were measured in plasma of tumor free and RXF393-bearing mice untreated (vehicle) or treated with sunitinib (9 days of treatments). Protocol of the study as in Fig. 6. Results are plotted as mean ±SD (n=4/group).

**B**, Murine IL-6 protein levels were measured in plasma of tumor-free and C26-bearing mice, untreated (vehicle) or treated with sunitinib (Protocol as in Fig.4). Results are plotted as mean ±SD (n=4/group).

Plasma was collected using EDTA as anticoagulant. IL-6 was measured by using Milliplex MAP human and mouse cytokine magnetic bead kit (Millipore), following manufacturer's instructions. Human VEGF was measured by ELISA (Quantikine®, R&D Systems), accordingly to manufacturer’s instructions. Each sample was analyzed in duplicate.
